# Shotgun proteomics as a viable approach for biological discovery in the Pacific oyster

**DOI:** 10.1093/conphys/cot009

**Published:** 2013-05-17

**Authors:** Emma Timmins-Schiffman, Brook L. Nunn, David R. Goodlett, Steven B. Roberts

**Affiliations:** 1 School of Aquatic and Fishery Sciences, University of Washington, Box 355020, Seattle, WA 98195, USA; 2 Genomic Sciences, University of Washington, Box 355065, Seattle, WA 98195, USA; 3 Medicinal Chemistry, University of Washington, Box 357610, Seattle, WA 98195, USA

**Keywords:** *Crassostrea gigas*, Pacific oyster, proteomics, tandem mass spectrometry

## Abstract

Shotgun proteomics offers an efficient means to characterize proteins in a complex mixture, particularly when sufficient genomic resources are available. In order to assess the practical application of shotgun proteomics in the Pacific oyster (*Crassostrea gigas*), liquid chromatography coupled with tandem mass spectrometry was used to characterize the gill proteome. Using information from the recently published Pacific oyster genome, 1043 proteins were identified. Biological samples (*n* = 4) and corresponding technical replicates (three) were similar in both specific proteins identified and expression, as determined by normalized spectral abundance factor. A majority of the proteins identified (703) were present in all biological samples. Functional analysis of the protein repertoire illustrates that these proteins represent a wide range of biological processes, supporting the dynamic function of the gill. These insights are important for understanding environmental influences on the oyster, because the gill tissue acts as the interface between the oyster and its environment. *In silico* analysis indicated that this sequencing effort identified a large proportion of the complete gill proteome. Together, these data demonstrate that shotgun sequencing is a viable approach for biological discovery and will play an important role in future studies of oyster physiology.

## Abstract (precis)

The oyster gill proteome (expressed proteins) was sequenced using shotgun proteomics. This effort represents the first time that a global, non-gel based approach has been used to characterize proteins from oyster gill. The data provide insight into the dynamic functions of this tissue and demonstrate the viability of this approach.

## Introduction

Fluctuations in gene and protein expression can be sensitive and specific indicators of biological processes. At the transcript level, several methodologies can be used to characterize expression from the gene-centric to systems level, including quantitative PCR (e.g. Griffitt *et al.*, 2006; Stumpp *et al.*, 2011), microarrays (e.g. Todgham and Hofmann, 2009; Lockwood *et al.*, 2010), and high-throughput sequencing (e.g. Polato *et al.*, 2011; Philipp *et al.*, 2012). The use of ­high-throughput sequencing technology has exponentially increased available genome and transcript information for taxa of ecological interest in recent years. While these results provide an accurate portrayal of changes at the molecular level, it is common that proteins have a more direct role in regulating physiological processes and responding to environmental change.

Historically, there have been several technical and analytical challenges in characterizing global protein expression. One challenge is the need to have sufficient genomic resources available to describe proteins of interest. Specifically, protein sequencing generally produces short amino acid fragments that require a known corresponding gene for identification and annotation purposes. However, lack of genomic resources has not completely hampered proteomic studies. For example, researchers characterized the physiological response of *Gillichthys mirabilis* gill tissue exposed to osmotic and temperature stress using two-dimensional gel electrophoresis without sequencing proteins (Kültz and Somero, 1996). In another study, researchers used surface enhanced laser desorption/ionization and identified 11 differentially expressed proteins in the gill tissue of *Oncorhynchus mykiss* exposed to zinc stress (Hogstrand *et al.*, 2002). Four proteins were identified based on a combination of their physical properties (i.e. mass and binding) coupled with sequence similarity comparisons with the limited number of teleost protein sequences in the SwissProt database (Hogstrand *et al.*, 2002).

The use of predicted protein sequences in closely related species can assist in annotation, but species-specific information will provide more accurate results. This is evident in a study on protein expression in pea (*Pisum sativum*) chloroplasts, where concurrent complementary DNA sequencing facilitated the identification of a greater number of proteins compared with identification through homology searches with closely related model species (Bräutigam *et al.*, 2008). The reason that species-specific information provides such an advantage is due to how modern-day protein sequence identification is executed. The vast majority of high-throughput mass spectrometry (MS) proteomics is accomplished by matching observed peptide fragmentation patterns (tandem mass spectra) to theoretical spectra. This is possible because peptides fragment in a predictable manner, allowing for theoretical tandem mass spectra to be created *in silico* from a given protein sequence, stressing the importance of the database used. These correlation-based algorithms require the peptide mass (precursor mass) and peptide fragmentation (tandem mass spectrum). Even when employing databases of closely related species, a large number of viable tandem mass spectra of peptides might not be assigned accurately to a protein, because a single amino acid mutation could significantly alter the peptide mass and resulting fragmentation pattern.

As technological advances have continued to increase the accessibility of whole transcriptomes and genomes to researchers, there is increasing interest in leveraging these data to carry out proteomic studies for both biological discovery and for better characterizing physiological responses to environmental change. Recently, the Pacific oyster (*Crassostrea gigas*) genome was sequenced (Zhang *et al.*, 2012). Given the availability of this resource, our objective was to quantify the level of information (and respective variability) attainable in proteomic studies in oysters. There have been a several prior studies examining protein expression in oysters using liquid chromatography coupled with tandem mass spectrometry (LC-MS/MS) with samples separated by two-dimensional gel electrophoresis (2-DE) beforehand. In larval oysters, these proteomic techniques have identified specific proteins that are responsible for early developmental changes in *C. gigas* (Huan *et al.*, 2012) and the larval *C. gigas* response to elevated partial pressures of CO_2_ (ocean acidification; Dineshram *et al.*, 2012). These methods have also been used to identify and sequence proteins that are differentially regulated in a range of physiological situations in adult oyster species. The discoveries include the following: the up-regulation of antioxidant proteins in response to ocean acidification (Tomanek *et al.*, 2011); expression profiles denoting high-quality oocytes (Corporeau *et al.*, 2012); differing proteomic profiles between disease-resistant and disease-susceptible oysters (Simonian *et al.*, 2009); and specific responses to metal exposure (Thompson *et al.*, 2011, 2012a, 2012b; Liu and Wang, 2012) and acid sulfate run-off (Amaral *et al.*, 2012). These seminal studies in marine invertebrate proteomics demonstrate that analysis of global protein expression is a powerful tool to facilitate our understanding of the molecular physiological response to environmental stressors.

An alternative to 2-DE approaches is to perform shotgun proteomics. Shotgun proteomics is the sequencing of a complex mixture of peptides using LC-MS/MS without prior separation (i.e. 2-DE). One of the main advantages of using 2-DE methods is that information on the physical properties of the proteins (mass and isoelectric point) can be used in the protein identification, whereas these empirical data are lost in the strictly tandem MS approaches. However, tandem MS has significantly greater data efficiency than gel-based approaches. The use of shotgun proteomics allows for a greater number of proteins to be identified rapidly from a single sample, providing a more complete metabolic picture of cellular function and physiology. This method has been demonstrated by Muralidharan *et al.* (2012), who used shotgun proteomics to uncover *Saccostrea glomerata* haemocyte proteomic responses to metal contamination, and by Dheilly *et al.* (2012, 2013), who explored the proteomic response of coelomocytes to immune challenge in two urchin species.

In this study, we used shotgun proteomics to sequence the gill proteome of the Pacific oyster, *Crassostrea gigas*. The gill is the interface between bivalves and their environment, necessitating that the tissue performs a variety of physiological functions in response to the environment (e.g. David *et al.*, 2007; Wang *et al.*, 2010). The identification of proteins that are expressed in gill tissue supports the development of tools that can help to guide future research on the molecular physiology of molluscs faced with stresses such as climate change and disease. The goal of this study was to determine the effectiveness of using a shotgun proteomics approach and to carry out functional characterization of proteins expressed in gill tissue.

## Materials and methods

### Oysters

Pacific oysters (*C. gigas*, 18 months old) were collected in Shelton, WA, USA. Oysters were transferred to Friday Harbor Laboratories (Friday Harbor, WA, USA) into a flow-through system at 13°C for 6 weeks. Eight 4 l vessels containing six oysters each were kept in a water bath with seawater flowing through at 57.5 ml/min. Vessels were cleaned every other day with fresh-water and salt-water rinses. Oysters were fed Shellfish Diet 1800 (Reed Mariculture, Campbell, CA, USA). At the end of six weeks, gill tissue was removed from four oysters and immediately flash frozen in liquid nitrogen for proteomic analysis.

### Protein digestion and desalting

Gill tissue samples (50–100 mg) were homogenized in 50 mM NH_4_HCO_3_ (100 µl) using RNAse-free plastic pestles. Each homogenized gill sample was sonicated four times with a probe sonicator and stored on dry ice between sonications. After sonication, protein concentrations were measured using the Bradford assay, following the manufacturer's protocol (Pierce, Thermo Fisher Scientific, Rockford, IL, USA). Urea (36 mg) was added to each sample (for a total concentration of 6 M) to denature and solubilize peptides. Next, 1.5 M Tris (pH 8.8; 6.6 µl) was added, followed by 200 mM (*tris*(2-carboxyethyl)phosphine) (2.5 µl). Samples were incubated for 1 h at 37°C on a shaker. To alkylate the proteins, 200 mM iodoacetamide (20 µl) was added. Samples were then vortexed, and incubated for 1 h at room temperature in the dark. To absorb excess iodoacetamide, 200 mM dithiolthreitol (20 µl) was added, followed by vortexing and incubation at room temperature for 1 h. A volume equal to approximately 100 µg was removed, and the remainder was discarded. Ammonium bicarbonate (200 µl of 25 mM) was added to dilute the urea, and then high-pressure liquid chromagography (HPLC) grade MeOH (50 µl) was added to each tube. Trypsin was solubilized in a trypsin dilution buffer (20 µl) to a concentration of 1 µg/μl (Promega, Madison, WI, USA), and 3 µl of this solution was added to each sample to digest the proteins enzymatically. The samples were incubated overnight at 37°C. The next day, dilute formic acid was added, and the samples were evaporated on the speed vac to near dryness. Samples were reconstituted in 200 µl of 5% acetonitrile and 0.1% trifluoroacetic acid.

Samples were desalted by passage through a pre-prepared MacroSpin column, following the manufacturer's specifications (The Nest Group, Southborough, MA, USA). After desalting, the remaining solvent was evaporated using a speed vac.

### Liquid chromatography and tandem mass spectrometry

Mass spectrometry was performed at the University of Washington Proteomics Resource (Seattle, WA, USA). Samples were resuspended in 2% acetonitrile and 0.1% formic acid in water (100 µl). Samples were then vortexed to mix and spun down at 21130 × g for 10 min. The supernatant was aliquoted to autosampler vials. Nano LC separation was performed with a nanoACUITY system (Waters, Milford, MA, USA) interfaced to an LTQ Orbitrap XL mass spectrometer (Thermo Scientific, San Jose, CA, USA). Peptides were trapped on a 100 µm i.d. × 20 mm long pre-column packed with 200 Å (5 µm) Magic C18 particles (C18AQ; Michrom, Auburn, CA, USA). For separation, a 75 µm i.d. × 250 mm long analytical column with a laser pulled emitter tip packed with 100 Å (5 µm) Magic C18 particles (C18Q; Michrom) was used and analysed in positive ion mode. For each LC-MS/MS analysis, an estimated amount of 0.5 µg of peptides was loaded onto the pre-column at 2 µl/min in water/acetonitrile (98%/2%), with 0.1% (v/v) formic acid. Peptides were eluted using an acetonitrile gradient flowing at 240 nl/min, using a mobile phase consisting of the following components: Solvent C (water, 0.1% formic acid) and Solvent D (acetonitrile, 0.1% formic acid). The gradient programme was as follows: 0–1 min, Solvent C (98%) and Solvent D (2%); 1 min, Solvent C (90%) and Solvent D (10%); 90 min, Solvent C (65%) and Solvent D (35%); 91–101 min, Solvent C (20%) and Solvent D (80%); and 102–120 min, Solvent C (98%) and Solvent D (2%). Peptide spectra were acquired by scans in the Orbitrap followed by the ion trap.

### Data acquisition

High-resolution full precursor ion scans were acquired at 60 000 resolution in the Orbitrap over 400–2000 *m*/*z* while six consecutive tandem mass spectra were acquired by collision-induced dissociation in the linear ion trap (LTQ). The data-dependent ion threshold was set at 5000 counts for MS/MS, and the maximum allowed ion accumulation times were 400 ms for full scans and 100 ms for MS/MS measurements. The number of ions accumulated was set to 1E6 for Orbitrap scans and 1E4 for linear ion trap MS/MS scans. An angiotensin and neurotensin standard was run after every eight injections. Each sample was injected in triplicate in a novel randomized order.

### Protein identification and data analysis

Peptide sequence and corresponding protein identification for all mass spectra was carried out using SEQUEST (Eng *et al.*, 1994) and the *C. gigas* proteome version 9 (Zhang *et al.*, 2012, http://dx.doi.org/10.5524/100030). A DECOY database was created by reversing the *C. gigas* proteome and adding it to the forward database. This was completed in order to determine false positive matches of peptide spectra matching, and yielded a false discovery rate of ∼0.6%. Search parameters included trypsin as the assigned enzyme and a precursor mass accuracy of +3 Da. SEQUEST results were analysed using PeptideProphet and ProteinProphet in order to evaluate peptide matches statistically and assign protein probabilities (Nesvizhskii *et al.*, 2003). Only proteins with a probability of ≥0.9 (estimated false discovery rate of 0.6%), a minimum of two unique peptide hits within a single replicate, and a minimum of four total tandem mass spectral assignments in the combined technical and biological replicates were used in further characterizations described below.

In order to annotate corresponding proteins, the *C. gigas* proteome (version 9) was compared with the UniProtKB/Swiss-Prot database (www.uniprot.org) using Blastp with an e-value limit of 1 × 10^−10^. Associated gene ontology (GO) terms were used to classify sequences based on biological process, as well as to categorize genes into parent categories (GO Slim). Enrichment analysis was used to identify over-represented biological processes in the gill proteome compared with the entire proteome [Database for Annotation, Visualization and Integrated Discovery (DAVID), version 6.7; Huang *et al.*, 2009a, 2009b, http://david.abcc.ncifcrf.gov/]. The results of the enrichment analysis were visualized in REViGO (Reduce and Visualize Gene Ontology; Supek *et al.*, 2011, http://revigo.irb.hr/). Normalized spectral abundance factor (NSAF; Florens *et al.*, 2006) was used to calculate expression for each protein in each oyster. Technical replicates were pooled by taking the sum of total independent tandem mass spectra for each protein (SpC). For each protein, SpC was divided by protein length (*L*). The NSAF is calculated from SpC/*L* divided by the sum of all SpC/*L* values for the proteins for a particular oyster. Comparisons of proteins identified across biological samples were visualized using Venny (Oliveros, 2007).

The minimum number of peptides needed to be sequenced to optimize unique protein identifications was determined using an *in silico* approach. A list was constructed of all sequenced peptides and their matching protein identification. Redundancies were maintained in this list, so that if a certain peptide was sequenced multiple times it was included multiple times in the list. Randomized subsets of this list were generated using the sample function in R (R Development Core Team, 2009). The number of hypothetically sequenced peptides in these lists ranged from 500 to 70 000. A plot was generated to visualize the relationship between each sample size of randomly chosen peptides and the number of unique proteins identified.

## Results

### Liquid chromatography and tandem mass spectrometry

A combined total of 175 818 tandem MS spectra were generated across all four biological and three technical replicates using the Orbitrap mass spectrometer (Table 1). Expression values were comparable between biological replicates, with *r*^2^ ranging from 0.800 to 0.889 (Supplementary Data 1). A total of 54 521 unique peptides contributed to the identification of 2850 proteins, with a probability score threshold of 0.9 (Supplementary Data 2). Of these proteins, 1043 had at least two unique peptide hits and four tandem mass spectra in the combined replicates. The mean coverage of proteins by sequenced amino acids was 13.3%. Protein identifications for each injection, including protein probability scores, number of total and unique spectra, and peptide sequences, are provided in Supplementary Data 3. The NSAF values for each protein are provided in Supplementary Data 4.

**Table 1:** Summary of the number of peptides sequenced and proteins identified for each oyster (labelled A–D)

	Oyster			
	A	B	C	D
Peptides sequenced (total)	44 720	43 646	44 177	43 275
Technical replicate no. 1	16 112	15 390	15 611	15 180
Technical replicate no. 2	14 645	14 329	14 592	14 290
Technical replicate no. 3	13 963	13 927	13 974	13 805
Proteins identified (total)	923	959	883	875
Technical replicate no. 1	731	730	704	683
Technical replicate no. 2	722	729	685	667
Technical replicate no. 3	694	771	657	677
Proteins identified in all replicates	509	514	484	478

**Figure 1:**
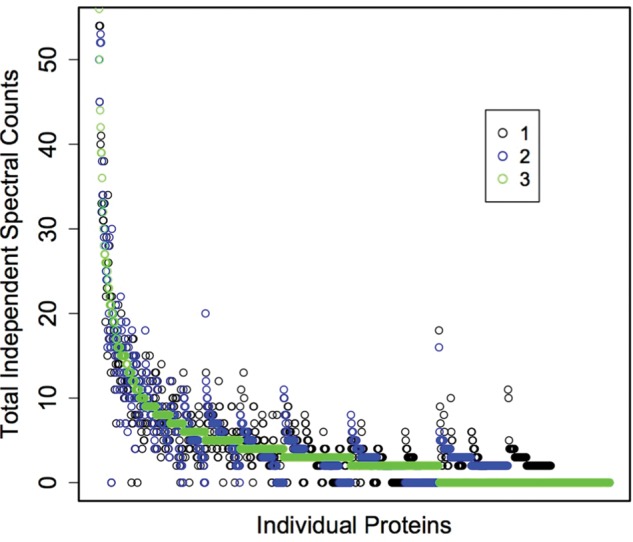
Total independent spectral counts for three technical replicates for oyster A plotted for each protein (*n* = 1500). Similar patterns were observed for the other three oysters (data not shown).

For all biological samples, the number of proteins identified in each technical replicate was consistent with minimal standard deviation (1.2–3.5%). In each biological replicate, the proteins were identified from between 43 275 and 44 720 sequenced peptides (standard deviation as a percentage of the mean ranged from 4.8 to 7.4%). For each oyster, 54–55% of the identified proteins were present in all three technical replicates. Using spectral counts as a proxy for relative expression, protein expression levels were consistent across technical replicates (Fig. 1).

**Figure 2:**
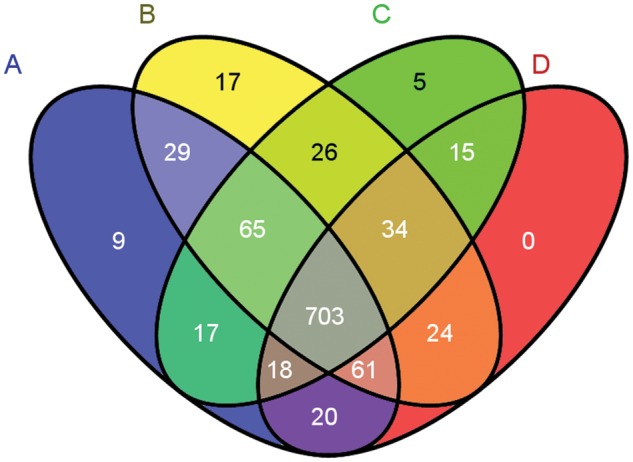
Venn diagram of proteins identified among biological samples. Proteins identified in oyster A are in the blue ellipse, B in yellow, C in green, and oyster D proteins are in the red ellipse.

The number of proteins identified in each oyster (after pooling technical replicates; see Methods) was 923, 959, 883, and 875 (Table 1). Most proteins (*n* = 703) were identified across all biological samples (Fig. 2).

In order to evaluate general protein expression and assess sample variability, the 10 most highly expressed proteins in each oyster were identified. There was not complete overlap in this group of proteins among the four oysters, so that a total of 12 proteins represent the most highly expressed for the entire dataset (Table 2). The 12 most abundant proteins across the four oysters analysed represent core cell structure and function, such as nucleosome assembly, cytoskeleton structure, muscle components, turnover of intracellular proteins, and protection against oxidative stress. Eight of these 12 proteins (arginine kinase, actin, histone H2A, histone H2B.3, histone H4, peptidyl-prolyl *cis-trans* isomerase, extracellular superoxide dismutase, and cytosol aminopeptidase) were identified in the top 10 most expressed proteins in all four oysters.

**Table 2:** The 12 most abundant proteins in the gill proteome as determined by identifying the 10 most abundant proteins in each oyster

Protein ID	Protein description	Accession no.	Oysters
CGI_10021481	Arginine kinase	O15990	A,B,C,D
CGI_10022730	Actin	O17320	A,B,C,D
CGI_10008058	Histone H2A	P02269	A,B,C,D
CGI_10008057	Histone H2B.3	P35069	A,B,C,D
CGI_10008056	Histone H4	Q28DR4	A,B,C,D
CGI_10025180	Peptidyl-prolyl *cis-trans* isomerase	P54985	A,B,C,D
CGI_10004092	Extracellular superoxide dismutase	Q08420	A,B,C,D
CGI_10006610	Cytosol aminopeptidase	Q65FE6	A,B,C,D
CGI_10013347	ATP synthase subunit β	Q05825	A,B,D
CGI_10012330	Tubulin β chain	P11833	B,C
CGI_10000082	Barrier-to-autointegration factor	Q6P026	C,D
CGI_10010974	Glyceraldehyde-3-phosphate dehydrogenase	P56649	A

Protein ID for the oyster is given, as well as protein description from UniProt-KB/SwissProt, SwissProt Accession Number for the homologous protein used to annotate the oyster protein, and the oysters in which the protein was detected.

**Figure 3:**
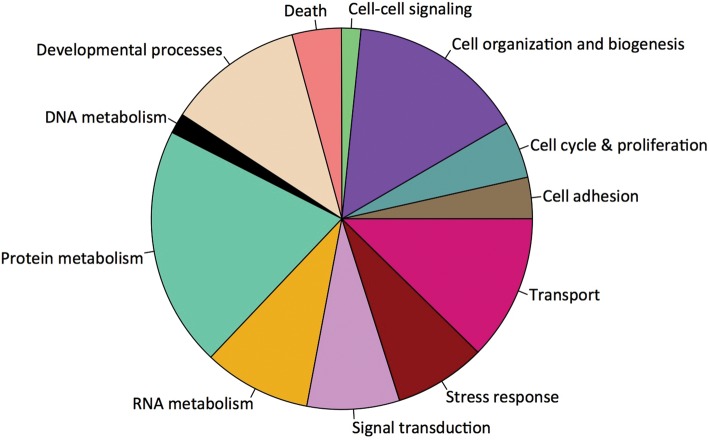
Representation of biological processes corresponding to the proteins identified from oyster gill tissue.

Of the 1043 proteins expressed across all samples, 1033 were annotated using the UnitProt-KB/SwissProt database. Of the annotated proteins, 888 were associated with Gene Ontology classifications. A majority of proteins were associated with the biological process of protein metabolism (*n* = 273), followed by cell organization and biogenesis (*n* = 201), and transport (*n* = 165) (Fig. 3).

Enrichment analysis was carried out to determine which biological processes were over-represented in gill tissue in comparison to the entire proteome. Several of the functional groups identified were associated with the abundant proteins involved in metabolism and transport, as well as structural processes (i.e. actin-filament and microtubule) and oxidation–reduction. The most significantly enriched biological process was generation of precursor metabolites and energy. Protein IDs (accession numbers starting with “CGI”) corresponding to the proteins that contributed to GO term enrichment are listed in Supplementary Data 5.

**Figure 4:**
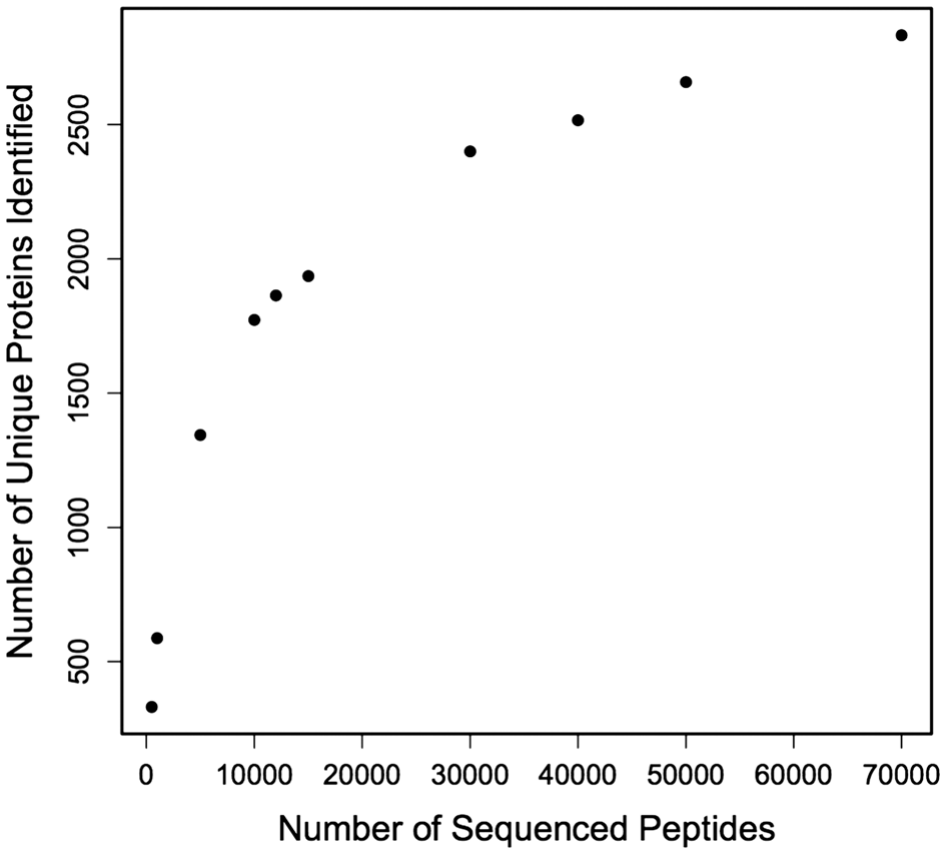
Predicted number of unique proteins that would be identified based on a sequential increase in peptide sequences.

The number of unique proteins identified with different numbers of sequenced peptides created an exponential curve (Fig. 4). The plateau began around 30 000–40 000 sequenced peptides, with a total of 2400–2516 unique peptides identified. New unique peptides were still identified in larger sample sizes of peptides, but the return per sequenced peptide diminished.

## Discussion

Technical and analytical challenges have resulted in limited focus on quantitative proteomics approaches in environmental physiology. Given the recent technological advances in the proteomics field (Yates *et al.*, 2009) and release of the Pacific oyster genome (Zhang *et al.*, 2012), we set out to assess the practical use of quantitative proteomics in this model species. For all biological samples, a majority of the proteins identified (54–55%) were present in all respective technical replicates. Relative expression across technical and biological replicates was also consistent (Fig. 1, Supplementary Data 1). However, there were some proteins not identified in all technical replicates. Thus, proteins with limited expression might not be detected and/or expression levels might not be reflected accurately. It should be noted that the inclusion of proteins in our analysis is highly dependent on threshold selection. In the present study, a protein was included only if it had two unique spectral hits within a replicate and had four total spectra across the combined technical replicate data. If the threshold were adjusted to be more conservative (i.e. a greater total spectral count threshold), variability would be reduced. With a total spectral count threshold of five, 983 proteins are identified and 56–57% of the proteins are in all three technical replicates; with a threshold of 10, 845 proteins are identified and 61–63% of the proteins are in all technical replicates (data not shown).

The number of proteins identified and subsequently annotated can vary tremendously based on experimental design, target tissue, match thresholds, and genomic resources available. In the present study, the majority of the proteins (703) were identified in all biological samples. Based on *in silico* analysis (Fig. 4), we have sequenced a relatively complete proteome for oyster gill tissue. In a study of European whitefish, *Coregonus lavaretus*, proteomics on fish larvae yielded sequencing of peptides corresponding to 1500 proteins (Papakostas *et al.*, 2012). The similar number of protein identifications in whitefish compared with our study (1043) is likely to be associated with the tissue complexity. In the whitefish study, whole body tissue was examined. In a metaproteomics study of marine microbes, 2273 distinct proteins were identified across 10 samples (Morris *et al.*, 2010). The large number of proteins identified by Morris *et al.* (2010) is evidence of the large number of organisms and ecological niches that were sampled in their study. Previous proteomics studies on Sydney rock oyster haemolymph have found relatively few proteins compared with the present study in gill tissue, with the number of identified proteins ranging from 49 to 514 (Simonian *et al.*, 2009; Thompson *et al.*, 2011, 2012a, 2012b; Muralidharan *et al.*, 2012). The identification of fewer proteins in haemolymph is probably because there are fewer cell types present in haemolymph compared with the gill.

In addition to assessing the feasibility of shotgun proteomics in the Pacific oyster, we were also able to provide a functional characterization of the gill proteome. Gene ontology characterization identified a majority of proteins associated with protein metabolism, cell organization and biogenesis, and transport (Fig. 3). These biological functions would be expected, because gill tissue is the primary interface between the oyster and the environment (water), where the tissue's major functions include ion regulation, respiration, and sorting of food particles. The high number of proteins involved in these GO categories is not necessarily unique to gill tissue but is likely to reflect the multifunctional nature of a tissue that responds to variable environments.

Enrichment analysis was performed to identify which functional groups of proteins expressed in gill tissue were over-represented incomparison to the complete protein repertoire. Several of the functional groups identified were associated with the abundant proteins involved in metabolism and transport, as well as cellular structure. These enrichment analysis findings are consistent with a previous transcriptomic comparison between *C. gigas* gill tissue and other tissues, with genes predominantly expressed in the gill being involved in epithelia morphogenesis, cilia movement, and detoxification and defense (Dheilly *et al.*, 2011). Some of the cytoskeletal proteins identified in gill were tektin-3, microtubule-associated protein futsch, and actin. Tektin is part of cilia and flagellar microtubules and has been found to change expression in response to an elevated partial pressure of CO_2_ (Dineshram *et al.*, 2013), and has also been identified in Sydney rock oyster haemolymph (Thompson *et al.*, 2012b). Transport proteins included ATP synthases and v-type proton ATP synthase. ATP synthase is a good marker of environmental stress in *C. gigas*, because its transcript expression is altered in response to hypoxia (David *et al.*, 2005) and pesticide exposure (Tanguy *et al.*, 2005). The most significantly enriched biological process was generation of precursor metabolites and energy. Many of the proteins that contributed to the over-representation of this GO category in the gill tissue are involved in metabolic processes, such as 2-­oxoglutarate dehydrogenase, dihydroplipoyllisin-residue acetyltransferase, glycogen phosphorylase, triose phosphate isomerase, and hexokinase. These enzymes are all involved in the breakdown of carbohydrates and other food inputs, and thus underline the important metabolic processes that occur in the gill.

Proteins involved in oxygen metabolism and reactive oxygen species defense were also enriched in gill tissue, providing further support for the importance of gill tissue in response to environmental change. Previous transcriptomics-based studies of oysters support that the oxidative stress response plays an important role in the gill tissue (e.g. David *et al.*, 2007; Fleury and Huvet, 2012). Genes and proteins responding to production of reactive oxygen species increase in oysters in many instances of environmental stress, such as exposure to contaminants (e.g. David *et al.*, 2007; Muralidharan et al., 2012), as well as exposure to ocean acidification (Tomanek *et al.*, 2011) and temperature stress (Meistertzheim *et al.*, 2007). Specific proteins that contribute to reactive oxygen species defense are enzymes instrumental in the physiological response to oxidative stress, such as the antioxidants superoxide dismutase, peroxiredoxin, and catalase.

The success of the shotgun sequencing effort was due in part to the recent publication of the *C. gigas* genome, ­emphasizing that the dissemination of genomic resources ­provides invaluable opportunities for advancement for the scientific community. The sharing of these large data sets, such as the genome and the gill proteome, will support further research into the effects of environmental changes on the oyster in terms of both acclimatization and adaptation. The characterization of the scope of acclimatization and adaptation are instrumental in understanding how the Pacific oyster, an ecologically and economically important species, can respond to climate change at the physiological and population levels. These research results demonstrate that shotgun sequencing of oyster gill tissue is a viable approach for biological discovery and that it will be likely to play an important role in future studies on oyster physiology.

## Supplementary material

Supplementary material is available at *Conservation Physiology* online.

Supplementary Data
