# Supplementary material for: Shotgun proteomics as a viable approach for biological discovery in the Pacific oyster
Source: Conserv Physiol. 2013 May 17;1(1):cot009. doi: 10.1093/conphys/cot009 (PMC4732435; doi:10.1093/conphys/cot009)
Supplement: Supplementary Data [file supp_1_1_cot009__index.html]

Shotgun proteomics as a viable approach for biological discovery in the Pacific oyster — Supplementary Data 

# Shotgun proteomics as a viable approach for biological discovery in the Pacific oyster

## Supplementary Data

Supplementary Data

**Files in this Data Supplement:**

- Supplementary Data - Docx file
- Supplementary Data 1 - jpg file
- Supplementary Data 2 - xlsx file
- Supplementary Data 3 - xlsx file
- Supplementary Data 4 - xlsx file
- Supplementary Data 5 - xlsx file
