# Supplementary material for: Shotgun proteomics as a viable approach for biological discovery in the Pacific oyster
Source: Conserv Physiol. 2013 May 17;1(1):cot009. doi: 10.1093/conphys/cot009 (PMC4732435; doi:10.1093/conphys/cot009)
Supplement: Supplementary Data [file supp_cot009_cot009supp.docx]

SUPPLEMENTARY DATA

**Supplementary Data 1**: Correlations of log(NSAF) data between all oyster pairs. The 1:1 line is plotted in pink on each graph and the R^2^ value is provided in the upper lefthand corner of the graph. Also included is a histogram showing the frequency of proteins for each log(NSAF) value, which follows a normal distribution.

**Supplementary Data 2**: Proteins identified based on tandem mass spectra compared to the *Crassostrea gigas* proteome. The protein accession number is provided for each protein identified (n=2,850). When proteins could be annotated, SwissProt Accession Number (SPID), e-value for the BLASTp search, and gene description are provided. For all proteins, the total number of tandem mass spectra is provided.

**Supplementary Data 3**: Spectra, peptide, and corresponding protein information for all 12 injections (4 biological samples x 3 technical replicates). Sheets are labeled as oyster ID and technical replicate (i.e. B_03 is the third technical replicate for oyster B). Each protein search result includes an entry number, one or more protein accession numbers, the probability that the protein assignment is correct, the percent coverage of the protein by the sequenced peptides, the number of unique peptides (non-redundant) used to identify the protein, the total number of peptides used to identify the protein, and the peptide sequences. Some of the entries have multiple protein accession numbers because numbers correspond to proteins with the same or highly similar sequences.

**Supplementary Data 4**: Calculated normalized spectral abundance factors (NSAF) for each oyster. NSAF was calculated by dividing spectral counts for each oyster (SpC, summed across three technical replicates) by protein length (SpC/L) and then dividing SpC/L by the sum of all SpC/L for that particular oyster (Florens et al. 2006). Proteins included in this file satisfy the thresholds of at least two unique peptide hits in a biological replicate and at least four spectral counts across all biological replicates.

**Supplementary Data 5:** List of proteins associated with enriched Gene Ontology terms in the gill tissue compared to the entire oyster proteome. Protein accession numbers are associated with UniProt accession numbers as well as the enriched GO term(s).
