# Supplementary figures and images for: Shotgun proteomics as a viable approach for biological discovery in the Pacific oyster
Source: Conserv Physiol. 2013 May 17;1(1):cot009. doi: 10.1093/conphys/cot009 (PMC4732435; doi:10.1093/conphys/cot009)

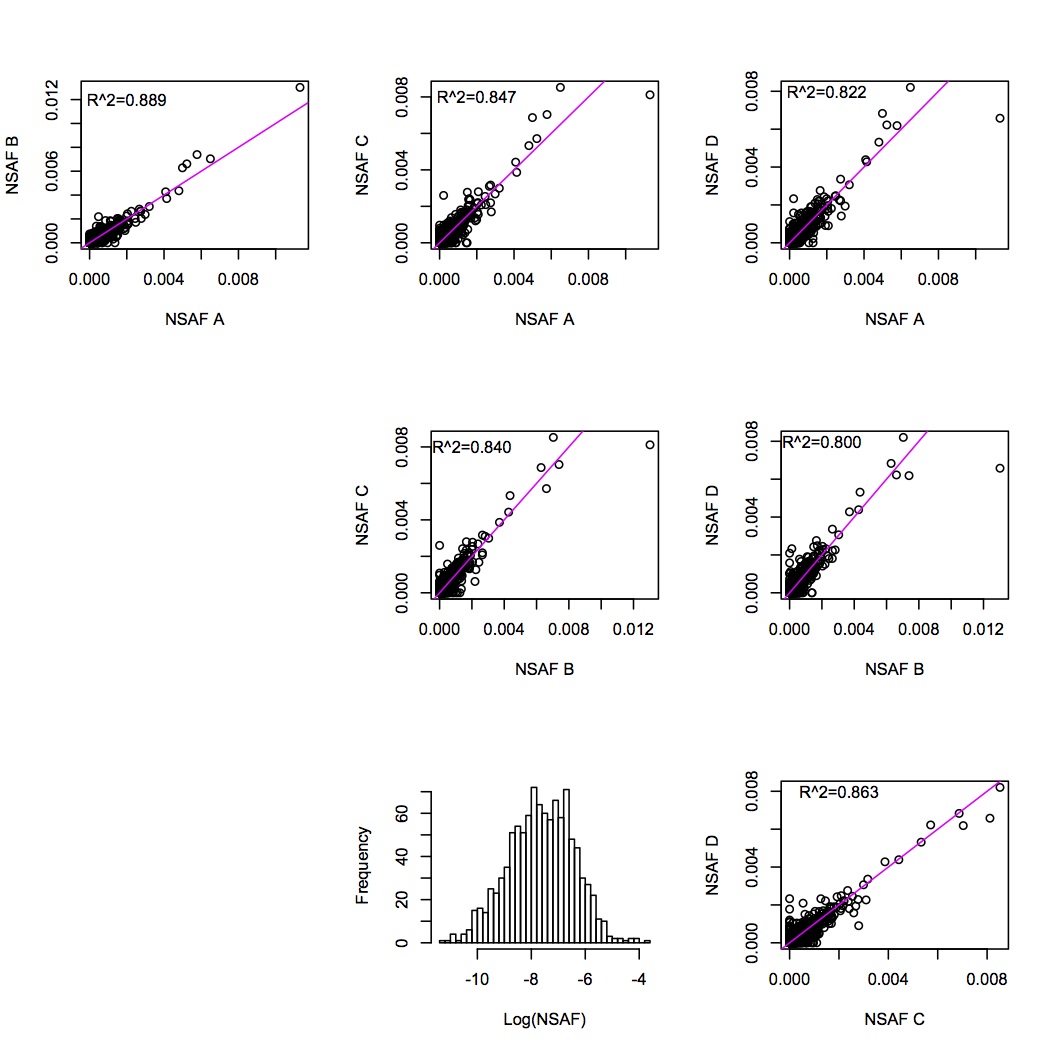

Supplement: Supplementary Data [file supp_cot009_cot009supp_data1.jpg]
